# Pyrolyzed deketene curcumin controls regulatory T cell generation and gastric cancer metabolism cooperate with 2-deoxy-d-glucose

**DOI:** 10.3389/fimmu.2023.1049713

**Published:** 2023-02-06

**Authors:** Takashi MaruYama, Hirofumi Miyazaki, Yun-Ji Lim, Jian Gu, Masaki Ishikawa, Taichi Yoshida, WanJun Chen, Yuji Owada, Hiroyuki Shibata

**Affiliations:** ^1^ Mucosal Immunology Section, National Institute of Dental and Craniofacial Research (NIDCR), National Institute of Health, Bethesda, MD, United States; ^2^ Department of Immunology, Graduate School of Medicine, Akita University, Akita, Japan; ^3^ Department of Organ Anatomy, Graduate School of Medicine, Tohoku University, Sendai, Japan; ^4^ Hepatobiliary Center, The First Affiliated Hospital of Nanjing Medical University and Research Unit of Liver Transplantation and Transplant Immunology, Chinese Academy of Medical Sciences, Nanjing, China; ^5^ Jiangsu Key Laboratory of Cancer Biomarkers, Prevention and Treatment, Collaborative Innovation Center for Cancer Personalized Medicine, Nanjing Medical University, Nanjing, China; ^6^ The Department of Pathology and Laboratory Medicine, Perelman School of Medicine, University of Pennsylvania, Philadelphia, PA, United States; ^7^ Department of Clinical Oncology, Graduate School of Medicine, Akita University, Akita, Japan

**Keywords:** tumor microenvironment, stomach neoplasms, adenosine triphosphate, glycolysis, regulatory T cells

## Abstract

Pyrolyzed deketene curcumin GO-Y022 prevents carcinogenesis in a gastric cancer mouse model. However, it is still less clear if GO-Y022 affects tumor-induced immune suppression. In this study, we found that GO-Y022 inhibited Treg generation in the presence of transforming growth factor beta 1 (TGF-β). However, GO-Y022 showed less impact on Foxp3^+^ Tregs in the gastric tumor microenvironment. Gastric tumor cells produce a large amount of L-lactate in the presence of GO-Y022 and diminish the inhibitory role of GO-Y022 against Treg generation in response to TGF-β. Therefore, naïve CD4^+^ T cells co-cultured with GO-Y022 treated gastric tumor cells increased Treg generation. GO-Y022-induced tumor cell death was further enhanced by 2-deoxy-d-glucose (2DG), a glycolysis inhibitor. Combination treatment of GO-Y022 and 2DG results in reduced L-lactate production and Treg generation in gastric tumor cells. Overall, GO-Y022-treatment with restricted glucose metabolism inhibits gastric tumor cell survival and promotes anti-tumor immunity.

## 1 Introduction

Gastric cancer is the third most common cause of cancer-related death worldwide, with approximately 780,000 global deaths in 2018 (1). Gastric cancer incidence hotspots exist in Eastern Europe and East Asia, including Japan (2). Salted food intake is a risk factor for gastric cancer development (3), whereas a high vegetable intake might reduce gastric cancer risk (3). Thus, adopting an anticancer diet aids in promoting gastric cancer therapy.

Transforming growth factor beta 1 (TGF-β1) induces Foxp3^+^ regulatory T cells (Tregs) (4), and TGF-β1 from gastric tumors inhibits anti-tumor immunity in the tumor microenvironment *via* this mechanism (5, 6). Anti-TGF-β1 treatment partly impairs Tregs’ generation in response to gastric cancer supernatants (7). In the gastric tumor microenvironment, Tregs produce huge quantities of interleukin (IL)-10 and prevent tumor immunity (8). Furthermore, Li et al. have demonstrated that the percentage of Foxp3^+^ Tregs in the gastric tumor microenvironment is correlated with the prognosis (9). Moreover, Tregs reduce T cell transendothelial migration in patients with gastric carcinoma (10). Therefore, TGF-β1-induced Treg generation in the tumor microenvironment is essential for regulating gastric anti-tumor immunity (11).

GO-Y022 (Deketomin^®^), a curcumin analog, is present in commercially available curry pastes and has anti-gastric tumor effects (12). We found that GO-Y022 prevents TGF-β1-induced Treg generation. Mechanistically, GO-Y022 inhibits the nuclear factor of activated T cell (NFAT) enrichment on the Foxp3 promoter and conserved non-cording sequence 1 region. Contrastingly, GO-Y022-treatment showed a minor effect on IL-10 production and suppressive ability of Tregs. In gastric tumors, GO-Y022 treatment controls gastric tumor cell proliferation and invasion by inducing metabolic changes. The role of glucose metabolism is crucial in the proliferation and cellular survival of gastric cancer (13). Therefore, a glycolysis inhibitor; 2-deoxy-d-glucose (2DG)-treatment prevents proliferation and promotes cell death in gastric tumor cells. Moreover, we found that a combination treatment of 2DG and GO-Y022 significantly inhibits the proliferation and death of gastric tumor cells. *In vivo* tumor models have demonstrated that GO-Y022-treatment has no significant impact on the Treg population in the gastric tumor microenvironment. GO-Y022 treated gastric tumor cells showed a higher production of L-lactate, a supporter of TGF-β-induced Tregs (14). GO-Y022 treatment of gastric tumor cells co-cultured with human CD4^+^ T cells increased Treg generation, which was significantly decreased in the presence of 2DG. Collectively, our findings suggest that GO-Y022 controls the gastric tumor cell metabolisms and tumor immunity cooperating with 2DG.

## 2 Material and methods

### 2.1 Experimental models

All experiments in this study were performed according to the guidelines approved by the Institutional Animal Care and Use Committee of Akita University, Akita, Japan, and the National Institute of Dental and Craniofacial Research (NIDCR), Bethesda, MD, USA. All methods were performed according to the relevant guidelines and regulations of Akita University and the NIDCR.

### 2.2 Mice

C57BL/6 and B6.SJL-Ptprc^a^ (CD45.1) mice were purchased from CLEA Japan, Inc. (Tokyo, Japan) and the Jackson Laboratory (Bar Harbor, ME, USA). K19-Wnt1/C2 mE (Gan) mice were bred by crossing K19-Wnt1 and K19-C2 mE mice (15). Mice aged 7–16 weeks used in this study were maintained in specific pathogen-free conditions at the animal facilities of Akita University and the NIDCR.

### 2.3 Enzyme-linked immunosorbent assay (ELISA)

ELISA kits for TGF-β1 (R&D systems, Minneapolis, MN) and the L-Lactate assay kit (Cayman Chemicals, Ann Arbor, MI, USA) were used to quantify the concentrations of these factors in the culture supernatants according to the manufacturer’s protocols. For the TGF-β1 ELISAs, the absorbance at 450 nm was read using a Spectramax Plus 384 plate reader (Molecular Devices, San Jose, CA). For the L-lactate ELISA, fluorescence was read at an excitation wavelength of 530 nm and an emission wavelength of 590 nm by Gen5 (Bio Tek, Winooski, VT).

### 2.4 Flow cytometry

Cells were fixed and permeabilized using the FOXP3 Staining Buffer Kit (eBioscience, San Diego, CA, USA) and were subjected to intranuclear FOXP3 staining according to the manufacturer’s instructions. Dead cells were stained using the Zombie Yellow™ Fixable Viability Kit (BioLegend, San Diego, CA, USA)or FITC-conjugated Annexin V (eBioscience) and propidium iodide (eBioscience) according to the manufacturer’s instructions. 2-NBDG (2-(*N*-(7-Nitrobenz-2-oxa-1,3-diazol-4-yl)Amino)-2-Deoxyglucose), Invitrogen™) were used to check glucose-uptake ability according to the manufacturer’s instructions. The cells were analyzed by flow cytometry using a BD LSRFortessa™ (BD Bioscience, San Jose, CA, USA), and the data were analyzed using FlowJo (Tree-Star version; Ashland, OR, USA).

### 2.5 Antibodies

PerCP-Cyanine5.5-conjugated anti-mouse CD45.1 (A20), PE/Cy7-conjugated anti-mouse/human Helios (22F6) and FITC-conjugated anti-mouse CD4 (GK1.5) were purchased from BioLegend; PE-conjugated and APC-conjugated anti-mouse Foxp3 antibodies (FJK-16S), FITC-conjugated anti-human CD4 (RPA-T4), eFluor660^®^-conjugated anti-human Foxp3 (PCH101) and Foxp3 monoclonal antibody (FJK-16s) were purchased from eBioscience; anti-Lamin A/C (346) antibody was purchased from Santa Cruz Biotechnology (Dallas, TX).; anti-SMAD3 (C67H9) antibody was purchased from Cell Signaling (Danvers, MA); anti-Phospho SMAD3 (phospho S423+S425) (C25A9) antibody was purchased from Abcam (Cambridge, UK); anti-α-tubulin (T-5168) antibody was purchased from Sigma Aldrich (Burlington, MA, USA).

### 2.6 Cell cultures

Naïve CD4^+^ T cells were isolated from mouse spleens using a mouse CD4^+^CD62L^hi^ T Cell Isolation Kit according to the manufacturer’s instructions (Miltenyi Biotec, Bergisch Gladbach, Germany). Purified cells (approximately 0.5 × 10^6^ cells/mL) were cultured at 37°C in Roswell Park Memorial Institute Medium (RPMI 1640; Lonza, Basel, Switzerland) containing 10% fetal bovine serum (Heat inactivated; GeminiBio,West Sacramento, CA), penicillin/streptomycin, and 50 μM 2-mercaptoethanol (Sigma Aldrich) with 1 μg/mL plate-bound anti-CD3 (eBioscience) and 1 μg/ml soluble anti-CD28 (eBioscience) for 1-3 days, as indicated in each experiment. Cyclosporine A (12.5 ng/ml, Sigma-Aldrich) was used as a NFAT inhibitor. Naïve CD4^+^ T cells were isolated from human PBMC using a naive CD4^+^ T Cell Isolation Kit II, according to the manufacturer’s instructions (Miltenyi Biotec). Purified cells (approximately 0.4 × 10^6^ cells/mL) were cultured at 37°C in X-VIVO™15 (Lonza, Walkersville, MD), 10% human AB serum (Sigma Aldrich) with Dynabeads human T-activator CD3/28 (Thermofisher Scientific) for 3 days. *In vitro* differentiation of mouse or human Foxp3^+^Tregs from naïve CD4^+^ T cells, 2 ng/ml or 0.2 ng/ml recombinant human TGF-β1 (PeproTech, Cranbury, NJ) in the presence or absence of 100 μM or 10 μM L-Lactate (Cayman Chemicals) were added. DMSO, 0.25 μM Curcumin (Sigma Aldrich) or 0.25 μM GO-Y022 (Nippon Carbide Industries Co., Inc., Tokyo, Japan) was used in T-cell cultures. SH-10-TC cells were maintained in RPMI1640 containing 10% fetal bovine serum and penicillin/streptomycin. GCIY cells were maintained in Minimum Essential Medium (Thermo-Fisher Scientific, Waltham, MA) containing 15% fetal bovine serum and penicillin/streptomycin (Sigma-Aldrich).

### 2.7 Real-time polymerase chain reaction (RT-PCR)

Total RNA was extracted using the RNeasy Mini Kit (Qiagen, Venlo, Netherlands), followed by cDNA synthesis using the High-Capacity cDNA Reverse Transcription Kit (Applied Biosystems). The resulting cDNA was evaluated by quantitative PCR (qPRC) using an Applied Biosystems 7500 RT-PCR system (Thermo-Fisher Scientific) or QuantStudio3 (Thermo-Fisher Scientific) instrument and TaqMan Gene Expression Master Mix (Thermo-Fisher Scientific). The primer pairs used for qPCR are listed in **Table S1**.

### 2.8 Suppression assay

CD4^+^CD25^+^ Tregs were isolated using the mouse CD4^+^CD25^+^ T Cell Isolation Kit according to the manufacturer’s instructions (Miltenyi Biotec, Bergisch Gladbach, Germany). Moreover, CD8^+^ T cells were isolated using the mouse CD8^+^ T Cell Isolation Kit according to the manufacturer’s instructions (Miltenyi Biotec). CD4^+^CD25^+^ T cells were cultured with 1 μg/mL plate-bound anti-CD3, 1 μg/ml soluble anti-CD28 and 10 ng/mL human IL-2 with or without 0.25 μM GO-Y022 for 3 days. Naïve CD8^+^ T cells (Responder cells) were isolated from CD45.1 mice and were labeled with CellTrace™ violet dye (Thermo-Fisher Scientific) (1:1000 dilution in PBS, 37°C for 20 min in dark) according to the manufacture’s institution. CellTrace™ violet dye-labeled naïve CD8^+^ T cells (0.8 × 10^5^ cells) were then cultured in a 96-well plate with Dynabeads™ T-activator CD3/CD28 (Veritask, Tokyo, Japan) in the presence or absence of CD4^+^CD25^+^ T cells.

### 2.9 Chromatin immunoprecipitation (ChIP) assay

Naïve CD4^+^ T cells were activated in culture with plate-bound anti-CD3 (1 μg/mL) and soluble anti-CD28 (1 μg/mL) for 24 h along with human TGF-β1 (2 ng/mL) in the presence or absence of GO-Y022 (0.25 μM). ChIP was performed using anti-NFATc1 binding protein (7A6) (Santacruz Biotechnology) and normal mouse IgG1 (G3A1) (Cell Signaling) using the iDeal ChIP-qPCR Kit (Diagenode, Denville, NJ, USA). In brief, cultured T cells were fixed using 1% formaldehyde (EMS diasum, Hatfield, PA, USA) and sonicated for 30 cycles (30 sec ON and 30 sec OFF at high power) at 4°C using Bioruptor Plus (Diagenode). Input and immunoprecipitated DNA were analyzed using SYBR™ Green PCR Master Mix (Applied Biosystems, Waltham, MA, USA) by qPCR using QuantStudio3 (Thermo-Fisher Scientific). Then, Fold enrichment (NFATc1/IgG) was calculated based on “percent input” in each sample. The following primer pairs were used for qPCR experiments: for the Foxp3 promoter: 5′-AGTGGCAGAGAGGTATTGAG-3′ and 5′-CCAAAGTCCTTACCTGGAGT-3′ and for the Foxp3 CNS1: 5′-TGGCTTCCAGTCTCCTTTAT-3′ and 5′-GACTTGAGTTGAGGCTAGGT-3′.

### 2.10 Reporter assay

EL4/LAF cells were transfected with pGL4-mouse Foxp3 promoter (−1702 to +174) (16) or pGL-4 basic using the nucleofector^®^ kit L with Amaxa Nucleofector™ II (Lonza) according to the manufacturer’s instructions. Luciferase activity was measured using TriStar2 LB942 (Berthold Technologies GmbH & Co. KG, Bad Wildbad, Germany) with the Duo-Luciferase Assay Kit (Genecopoeia, Rockville, MD, USA) according to the manufacturer’s instructions.

### 2.11 Nuclear extraction

Nuclear and cytosol fractions from cultured naïve T cells (2h, 2 ng/ml TGF-β1 ± 0.25 μM GO-Y022) were collected using Nuclear Extract Kit (Active motif, Carlsbad, CA) according to the manufacturer’s instructions.

### 2.12 Extracellular flux assay

The oxygen consumption rate (OCR) of tumor cells was measured using a Seahorse Bioscience XF^®^96 Extracellular Flux Analyzer (Agilent Technologies, Santa Clara, CA) according to the manufacturer’s instructions (17). Tumor cells were plated at approximately 30,000 cells/well in Seahorse 96-well plates 24 h before the assay. Tumor cells were treated with 0.007 μM dimethyl sulfoxide (DMSO) or 5 μM GO-Y022 for 3 h; then, the OCR and extracellular acidification rate were measured using the XF Cell Mito Stress Test Kit, XF cell energy stress kit and the Glycolytic rate test kit, respectively. ATP production was calculated by the decrease in OCR upon injecting 1 μM oligomycin (ATP synthase inhibitor) representing the portion of basal respiration (**Figure S1**).

### 2.13 shRNA knockdown experiments

A total of 1 ×10^6^ EL4/LAF lymphoma cells were transfected with 1 ug NFATc1 shRNA (Santa Cruz Biotechnology, #B1709) or control shRNA (Santa Cruz Biotechnology) by using Plasmid Transfection Reagent (Santa Cruz Biotechnology, sc-108061). A fresh medium added 24 h after transfection and incubated for another 48h. Subsequently, the cells were stimulated by using Dynabeads™ mouse T cell activator (CD3/CD28) in the presence of 2 ng human TGF-β. The *Foxp3* expression was checked 8h after stimulation by using real time PCR.

### 2.14 Gan mouse model treated with GO-Y02*2*

Gan mice (9 weeks old) were fed a 5-g high-fat diet 32 alone or high-fat diet 32 with 0.5% (*weight*/*weight*) GO-Y022 every day for 7 weeks (12), before the gastric tumor cells were taken and fixed with paraformaldehyde. This experiment was performed according to the guidelines set by Akita University (#a-1-2641). Stomachs of 9–16week-old Gan mice (fed with 0.5% GO-Y022 high fat diet) were fixed using 10% neutral buffered formalin, washed using a series ethanol solution (70-100%) and then embedded in paraffin. Immunohistochemistry was performed by Biopathology Institute Co., Ltd (Oita, Japan). To summarize, ethanol was used to remove the paraffin on the slide, and antigen retrieval was performed by autoclaving these samples at 120°C for 10min. Primary antibodies (anti-Foxp3 [FJK-16s; eBioscience] or anti-CD8a [D4W2Z; Cell Signaling TECHNOLOGY]) were applied to these samples and left at 4°C for overnight. Secondary antibodies (Rabbit anti-Rat IgG H&L (Biotin) [Ab6733; Abcam] or Simple stain mouse MAX-PO [Nichirei414341, NICHIREI Biosciences Inc., Tokyo, Japan]) were then applied to the samples and left at room temperature for 30 min. The samples were stained with 3,3’ diaminobenzidine identify the positive population. Then hematoxylin staining was performed to identify the nuclei.

### 2.15 Cytotoxicity assay

SH-10-TC cells were cultured for 24 h in the presence or absence of 5 μM GO-Y022 or 5 mM 2DG. GCIY cells were cultured for 24 h in the presence or absence of 10 μM GO-Y022 or 5 mM 2-deoxy-glucose (2DG). Cytotoxicity was measured using Cyto Tox96^®^ (Promega, Madison, WI) according to the manufacturer’s instructions. The absorbance at 490 nm was measured using a Spectramax Plus 384 plate reader.

### 2.16 Live cell-counting assay

Naïve CD4^+^T cells were cultured for 20 h in the presence or absence TGF-β, 0.25 μM curcumin, or 0.25 μM GO-Y022. One-tenth of the volume of the Cell-Counting Kit 8 reagent (Apexbio Technology LLC, Houston, TX, USA) was added to assess the dehydrogenases activity in live cells. After 4 h of incubation at 37°C and 5% CO_2_, WST-8 formazan orange dyes (O.D. 450 nm) is generated by dehydrogenases in the numbers of live cells and the absorbance was measured at 450 nm using a Spectramax Plus 384 plate reader (Molecular Devices, San Jose, CA). In case of Tregs, splenic CD4^+^CD25^+^ Treg cells were cultured with 1 μg/mL plate-bound anti-CD3 and 1 μg/ml soluble anti-CD28 in the presence of 10 ng/mL hIL-2 with or without 0.25 μM GO-Y022 as indicated for 68 h, followed by the addition of the Cell-Counting Kit 8 reagent (4 h). n case of gastric tumor cell lines, SH-10-TC or GCIY cells were cultured for 24 h in the presence or absence of GO-Y022 or 2DG. One-tenth of the volume of the Cell-Counting Kit 8 reagent (Apexbio Technology LLC) was added, and after a 1-h incubation at 37°C and 5% CO_2_ and the absorbance (WST-8 formazan orange dyes: O.D. 450 nm) was measured using a Spectramax Plus 384 plate reader (Molecular Devices).

### 2.17 Co-culture systems

For purified human naïve CD4^+^ T cells, a human naïve CD4^+^ T cell isolation kit was used according to the manufacturer’s instructions (Miltenyi Biotec). Human naïve CD4^+^ T cells (1 × 10^5^ cells) and SH-10-TC cells (3 × 10^4^ cells) were cultured at 37°C in RPMI 1640 (Lonza) containing 10% fetal bovine serum (Heat inactivated; GeminiBio), penicillin/streptomycin, and 50 μM 2-mercaptoethanol (Sigma Aldrich) in the presence of CD3/28 Dynabeads (Thermo-Fisher Scientific) for 48 h in the presence or absence of 5 μM GO-Y022 or 5 mM 2DG.

### 2.18 ATP detection assay

For ATP-release from SH-10-TC cells, ATP-detection assay kit - Luminescence was used according to the manufacturer’s instructions (Cayman Chemical). SH-10-TC cells (0.25 × 10^5^ cells/200 μl/wells) (96 well plate) were cultured in the presence or absence of 5 mM 2DG or 5 μM GO-Y022. After 24h of incubation at 37°C and 5% CO_2_, change the supernatants to PBS and incubate another 10 min at 37°C and 5% CO_2_. The luminescence according to ATP-release from tumor cells in each treatment (PBS-samples) was read using CentroXS^3^ LB960 (Berthold Technologies, Bad Wildbad, Deutschland).

### 2.19 Statistical analysis

All statistical analyses were performed using GraphPad Prism 5 (GraphPad Software). Unpaired Student’s t-test was used to compare the two groups. One-way analysis of variance with *post-hoc* Tukey’s multiple comparison test was used for comparison of multiple groups. Statistical significance was set at *p* < 0.05; *p < 0.05, **p < 0.01, and ***p < 0.001.

## 3 Results

### 3.1 Pyrolyzed deketene curcumin inhibits TGF-β-induced generation of Foxp3^+^ Tregs

**Figure 1:**
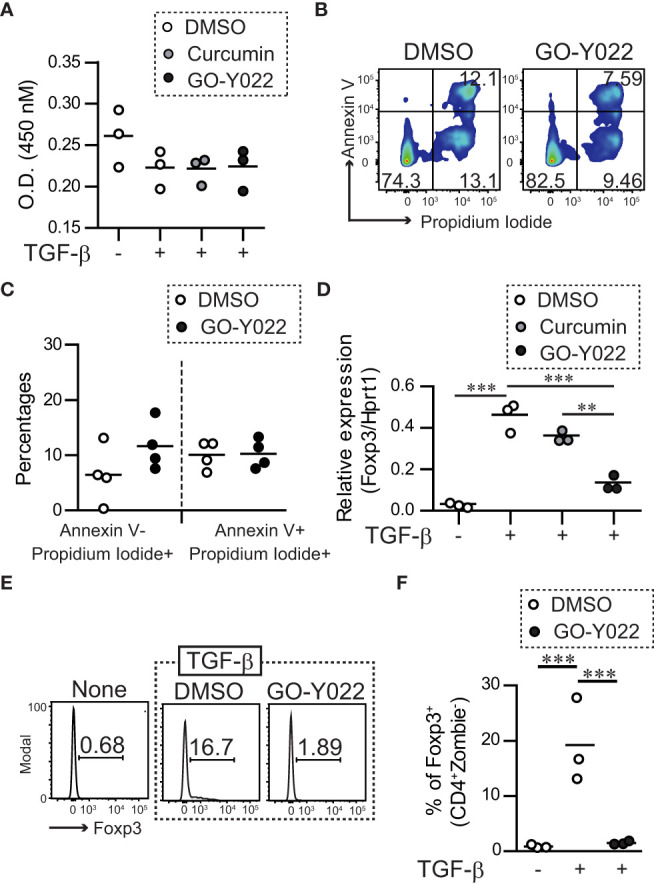
GO-Y022 inhibits TGF-β-induced generation of Tregs. **(A)** Live cell-counting assay. Naïve splenic CD4^+^ T cells were cultured with plate-bound anti-CD3 and soluble anti-CD28 in the presence or absence of 2 ng/mL human TGF-β1 and 0.007 μM DMSO (control), 0.25 μM curcumin or 0.25 μM GO-Y022 as indicated for 20 h, followed by the addition of the Cell-Counting Kit 8 reagent (4 h). The circles indicate independent experiments. The horizontal bars represent the mean. One-way analysis of variance (ANOVA) with *post-hoc* Tukey’s multiple comparison test was employed. **(B, C)** Flow cytometry analysis using live and dead staining (Annexin V and Propidium iodide). Purified naïve CD4^+^ T cells were cultured with plate-bound anti-CD3 and soluble anti-CD28 in the presence of 2 ng/mL human TGF-β1 and 0.007 μM DMSO or 0.25 μM GO-Y022 for 24 h and the CD4^+^ cell population was gated. The data show representative density plots **(B)**. Statistical analyses of apoptotic dead cells (Annexin V^+^Propidium Iodide^+^), early apoptotic dead cells (Annexin V^+^Propidium Iodide^+^) or necroptosis dead cells (Annexin V^-^Propidium Iodide^+^) were compared between 0.25 μM GO-Y022-treated and 0.007 μM DMSO-treated cultured CD4^+^ T cells (plate-bound anti-CD3 and soluble anti-CD28 in the presence of 2 ng/mL human TGF-β1). Data were pooled from four independent experiments **(C)**. **(D)** The real-time quantitative analysis results of 0.007 μM DMSO-, 0.25 μM curcumin-, or 0.25 μM GO-Y022 treated naïve CD4^+^ T cells for 24 h plate-bound anti-CD3 and soluble anti-CD28 in the presence or absence of 2 ng/mL human TGF-β1. The circles indicate independent experiments. The horizontal bars represent the mean. **(E, F)** Frequency of Foxp3^+^ Tregs in the entire CD4^+^ cell population. Naïve splenic CD4^+^T cells were cultured with plate-bound anti-CD3 and soluble anti-CD28 in the presence or absence of 2 ng/mL human TGF-β1 or 0.007 μM DMSO or 0.25 μM GO-Y022 as indicated for 3 days. The data are representative histogram **(E)**. Statistical analyses of the percentages of Foxp3^+^Tregs were compared between 0.25 μM GO-Y022-treated and 0.007 μM DMSO-treated cultured CD4^+^ T cells with plate-bound anti-CD3 and soluble anti-CD28 in the presence or absence of 2 ng/mL human TGF-β1. Data were pooled from four independent experiments **(F)**. The horizontal bars represent the mean. Student’s t-test **(C)** or one-way ANOVA with *post-hoc* Tukey’s multiple comparison test **(A, D, F)** was used. Statistical significance was set at *p* < 0.05; **p < 0.01, and ***p < 0.001.

Gastric tumor cells produce enormous quantities of TGF-β and induce Foxp3^+^Tregs (18), which negatively regulate anti-tumor immunity. As expected, the supernatants of the gastric tumor SH-10-TC cells produced a large amount of TGF-β1; (5 × 10^5^ cells/ml SH-10-TC cultured supernatants (72 h) = 0.81 ± 0.01 ng/ml; **Figure S8A**) and induced Foxp3^+^ Tregs generation *in vitro* (**Figures S2A, B**). As a high dose of GO-Y022 could induce apoptotic cell death in tumor cell lines (12), we first added GO-Y022 to cultured CD4^+^T cells and found that 0.25 μM GO-Y022 did not affect the cell viability (optical density [O.D.] 450 nM: DMSO treated = 0.222 ± 0.022, Curcumin treated = 0.225 ± 0.016, GO-Y022 treated = 0.223 ± 0.025) (**Figure 1A**) and apoptotic cell death in the presence of TGF-β (AnnexinV^+^ Propidium Iodide^-^ population; DMSO treated = 6.46% ± 5.21%, GO-Y022 treated = 11.66% ± 4.39%, p-value = 0.1780) (**Figures 1B, C**). Then, we found that 0.25 μM GO-Y022 treatment strongly decreased the TGF-β-induced mRNA levels (relative Foxp3/Hprt1 value: DMSO treated = 0.46 + 0.07, Curcumin treated = 0.36 + 0.02, GO-Y022 treated = 0.13 ± 0.03) (**Figure 1D**) and protein levels of Foxp3 expression (percentages of Foxp3 in CD4^+^ T cells: DMSO treated = 19.2 ± 7.66, GO-Y022 treated = 1.51 ± 0.32) (**Figures 1E, F**). Thus, 0.25 μM GO-Y022 prevents Foxp3^+^Tregs generation in response to TGF-β without the effects of cell death.

### 3.2 Pyrolyzed deketene curcumin inhibits NFAT binding to Foxp3 promoter and CNS1 in response to TGF-β

**Figure 2:**
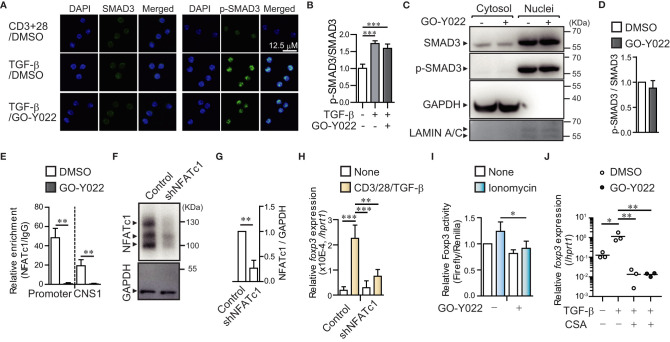
GO-Y022 prevents NFATc1 binding to *Foxp3* gene regulatory elements. **(A, B)** TGF-β/SMAD signaling pathway. Representative fluorescence microscopic images (Leica SP8; Leica, Wetzler, Germany) of SMAD3 (Green), phospho-SMAD3 (Green) and Nuclei (stained with DAPI; Blue) of three independent experiments. Naïve CD4^+^ T cells were stimulated with plate-bound anti-CD3, soluble anti-CD28 and 2 ng/mL human TGF-β1 in the presence or absence of 0.25 μM GO-Y022 for 2h **(A)**. Statistical analyses of phospho-SMAD3 (p-SMAD3)/SMAD3 expression (n = 6, mean with standard error of the mean) **(B)**. **(C, D)** Phosphorylation levels of SMAD3 in nuclear. Representative Western blotting image of SMAD3, p-SMAD3, and α-tubulin of three independent experiments. Naïve CD4^+^ T cells were stimulated with plate-bound anti-CD3, soluble anti-CD28 and 2 ng/mL human TGF-β1 in the presence or absence of 0.25 μM GO-Y022 for 2h **(C)**. Relative p-SMAD3/SMAD3 expression in nuclear fraction. Without GO-Y022 (DMSO-treatment) of p-SMAD3/SMAD3 was set as “1.”. The horizontal bars represent the mean and standard deviation. Data represent at least three independent experiments **(D)**. **(E)** RT-PCR documenting the relative amount of chromatin immunoprecipitation (NFATc1/IgG) at the Foxp3 locus (n = 3) using cultured naïve CD4^+^ T cells (24 h) with plate-bound anti-CD3, soluble anti-CD28 and 2 ng/mL human TGF-β1 with or without GO-Y022 and 0.007 μM DMSO. White bar represents DMSO; black bar represents 0.25 µM GO-Y022. The horizontal bars represent the mean and standard deviation. Data represent at least three independent experiments. **(F)** Western blotting by using EL4/LAF lymphoma cells which is transfected control or NFATc1 shRNA particle after 72h. Control of shRNA (NFATc1/GAPDH) was set as “1.”. The horizontal bars represent the mean and standard deviation (n=3). Data represent at three independent experiments **(G)**. **(H)** Real time PCR of *Foxp3* gene expression. The horizontal bars represent the mean and standard deviation (triplicate samples). Data represent at least three independent experiments. **(I)** A Foxp3 promoter assay was performed in EL4/LAF T lymphoma cells. Eighteen hours after plasmid transfection, the cells were incubated with 1 µM GO-Y022 or 0.007 μM DMSO in the presence or absence of 250 nM ionomycin. Without ionomycin and GO-Y022 stimulation was set as “1.” Data are pooled from three independent experiments (n = 3); the mean and standard deviation are shown. **(J)** The real-time quantitative analysis results of 0.007 μM DMSO- or 0.25 μM GO-Y022 treated naïve CD4^+^ T cells for 24 h plate-bound anti-CD3 and soluble anti-CD28 in the presence or absence of 2 ng/mL human TGF-β1, 12.5 ng/ml Cyclosporine A (CSA). The circles indicate independent experiments. The horizontal bars represent the mean. Student’s t-test **(D, E, G, H)** or one-way ANOVA with *post-hoc* Tukey’s multiple comparison test **(B, I, J)** was employed. Statistical significance was set at *p* < 0.05; *p < 0.05; **P<0.01 and ***P<0.001.

Next, we focused on the molecular mechanisms underlying the roe of GO-Y022 in controlling Treg generation. It is known that the TGF-β/SMAD axis plays a key role in initiating Foxp3^+^ Treg generation (19). However, GO-Y022 did not prevent the TGF-β/SMAD axis in cultured naïve CD4^+^ T cells (GO-Y022 treatment showed a 1.27 ± 0.27-fold increase in p-SMAD3 expression compared to the DMSO control) (**Figures 2A, B**), which was also observed in the nuclei (**Figures 2C, D**). Another signal molecule to control the *Foxp3* expression in CD4^+^ T cells; the transcription factor NFATc1 can enrich the *Foxp3* promoter and conserved CNS1 region and positively regulate *Foxp3* gene expression along with SMAD (20). We purified naïve CD4^+^ T cells and cultured them with TGF-β in the presence or absence of GO-Y022. Then we performed a chromatin immunoprecipitation assay to reveal the NFATc1 enrichment on the Foxp3 promoter and enhancer regions (**Figure 2E**). We found that GO-Y022 treatment significantly inhibited NFATc1 enrichment on the *Foxp3* promoter (relative value of NFATc1/IgG: DMSO treated = 48.4 ± 9.71, GO-Y022 treated = 0.66 ± 1.06) and conserved CNS1 (NFATc1/IgG: DMSO treated = 19.27 ± 6.26, GO-Y022 treated = 0.49 ± 0.52) (**Figure 2E**). Then, we addressed the role of NFATc1 and found that NFATc1 knockdown leads to a decrease in *Foxp3* gene expression in response to TCR and TGF-β stimulation (**Figures 2F–H**). Next, we performed a Foxp3 reporter assay for EL4/LAF T lymphoma cells, reported to express endogenous Foxp3 (20). We confirmed that 1 uM of GO-Y022 does not affect cell death in EL4/LAF T lymphoma cells (**Figure S3**). Furthermore, the NFAT activator ionomycin activates *Foxp3* reporter activity (1.23 ± 0.18-fold increase), prevented by GO-Y022 treatment (0.91 ± 0.13-fold increase) (**Figures 2I**, **S4**). Oppositely, the NFAT inhibitor cyclosporine A inhibits *Foxp3* expression, which did not further decrease *Foxp3* expression by GO-Y022-treatment (**Figure 2J**). Thus, GO-Y022 controls Treg generation *via* NFAT signaling.

### 3.3 GO-Y022 had little impact on the suppressive ability of Tregs in co-culture with CD8^+^ T cell

**Figure 3:**
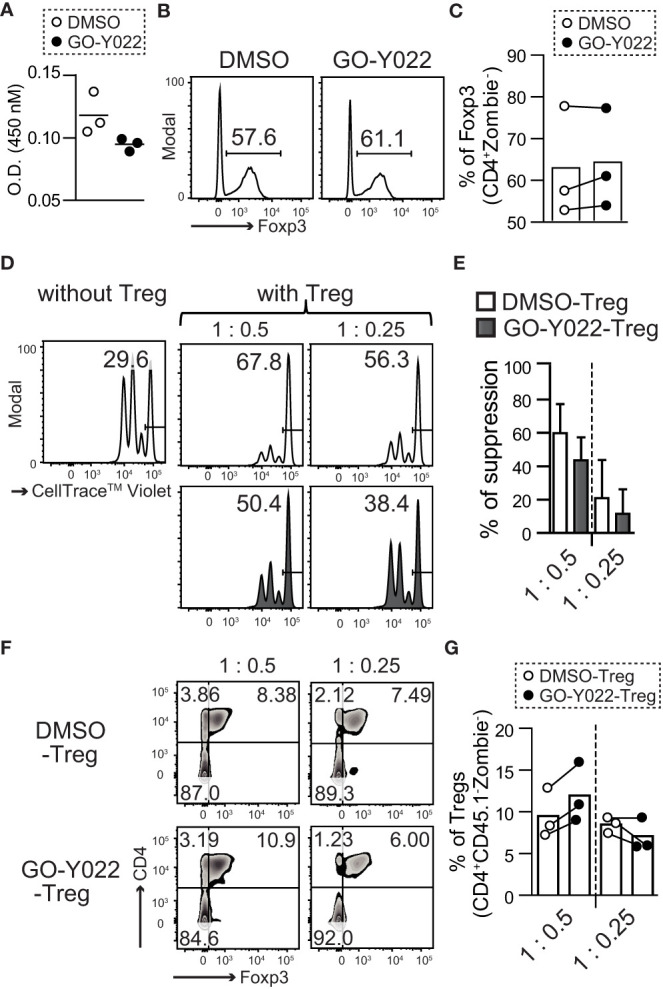
GO-Y022 has little effect on the immunosuppressive ability of Tregs. **(A)** Live cell-counting assay. Splenic CD4^+^CD25^+^ Treg cells were cultured in plate-bound anti-CD3, with soluble anti-CD28 and 10 ng/mL hIL-2 in the presence of 0.25 μM GO-Y022 or 0.007 μM DMSO as indicated for 68 h, followed by the addition of the Cell-Counting Kit 8 reagent (4 h). **(B, C)** The frequency of Foxp3^+^ Tregs in the entire CD4^+^ cell population. Splenic CD4^+^CD25^+^ Treg cells were cultured in plate-bound anti-CD3, with soluble anti-CD28 and 10 ng/mL hIL-2 in the presence of 0.25 μM GO-Y022 or 0.007 μM DMSO as indicated for 3 days. Data represent at least three independent experiments **(B)**. Statistical analyses of the percentage of Foxp3^+^Tregs were compared between 0.25 μM GO-Y022-treated and or 0.007 μM DMSO-treated cultured CD4^+^CD25^+^ Tregs (stimulated with plate-bound anti-CD3, soluble anti-CD28 and 10 ng/mL hIL-2). The circles indicate independent experiments and the horizontal bars represent the mean. Data were pooled from three independent experiments **(C)**. **(D, E)** The proliferation ratio of CellTrace™ Violet-labeled CD8^+^ T cells isolated from CD45.1 mice and cultured with or without CD4^+^CD25^+^ Tregs for 72 **(H)** Tregs were treated with 0.25 µM GO-Y022 (black fill) or 0.007 μM DMSO control (white fill) for 3 days before co-culture. The data showed a representative histogram gated on the CD8^+^CD45.1^+^Zombie^-^ population **(D)**. The relative suppressive ability of Tregs; the percentage of non-proliferative CD8^+^ T cells without Tregs is set to 0 (Leftmost figure in **D**). Data were pooled from three independent experiments. The mean and standard deviation is shown **(E)**. **(F, G)** Stability of Tregs. Foxp3 expression in CD4^+^CD25^+^ Tregs, which were co-cultured with CD8^+^ T cells isolated from CD45.1 mice for 72 **(H)** The data show representative density plots **(F)**. Statistical analyses of the percentage of Foxp3^+^Tregs were performed between 0.25 μM GO-Y022-treated and 0.007 μM DMSO-treated cultured CD4^+^CD25^+^ Tregs in the co-cultured systems. Data were pooled from three independent experiments. The circles indicate independent experiments and the horizontal bars represent the mean **(G)**. The graph shows the mean and standard deviation. Student’s t-test **(A, C, E, G)** was employed.

The proliferation, stability and suppression abilities of Tregs affect cancer patients’ prognosis (21). However, 2.5 μM GO-Y022 treatment showed a minor impact on the number of live cells (O.D. 450 nM: DMSO treated = 0.118 ± 0.016, GO-Y022 treated = 0.094 ± 0.005, p-value = 0.0831) (**Figure 3A**), proliferation (**S5A, B**) and stability (percentage of Foxp3 in CD4 ^+^ T cells: DMSO treated = 62.7% ± 13.2%, GO-Y022 treated = 64.1% ± 11.9%, p-value = 0.9008) of CD4^+^CD25^+^Tregs (**Figures 3B, C**). Helios is a transcriptional factor that may be involved in the stability of Tregs (22). However, Helios expression was not different between DMSO treated and GO-Y022 treated CD4^+^CD25^+^Tregs (**Figures S6A, B**). As Tregs in the tumor microenvironment are key to regulating CD8^+^T cells-mediated tumor rejection (23), we also investigated the suppression ability of GO-Y022-treated Tregs co-cultured with CellTrace™ Violet -labeled responder cells (CD8^+^ T cells). When responder cells were cultured without CD4^+^CD25^+^Tregs, approximately 70% of responder cells divided (**Figure 3D**). We also found that CellTrace™ Violet-labeled responder cells reduced the number of divisions co-cultured with CD4^+^CD25^+^Tregs, however the suppression abilities of GO-Y022-treated and DMSO-treated Tregs were not significantly different (percentages of suppression [T:Tregs = 1:0.5]: DMSO treated Tregs = 59.75% ± 17.75%, GO-Y022-treated Tregs = 43.56% ± 13.85%, p-value = 0.2781, [T:Tregs = 1:0.25]: DMSO treated Tregs = 20.8% ± 22.8%, GO-Y022-treated Tregs = 11.4% ± 14.8%, p-value = 0.5829) (**Figures 3D, E**). In this co-culture system, we found that the level of Foxp3 expression (reflects to stability and suppression ability of CD4^+^CD25^+^Tregs) did not differ significantly between the GO-Y022- and DMSO treated Tregs (percentages of Foxp3 in Tregs [T:Tegs = 1:0.5]: DMSO treated Tregs = 9.51% ± 2.98%, GO-Y022-treated Tregs = 11.98% ± 3.60%, p-value = 0.4162, [T:Tregs = 1:0.25]: DMSO treated Tregs = 8.51% ± 0.94%, GO-Y022-treated Tregs = 7.07% ± 1.97%, p-value = 0.3209) (**Figures 3F, G**). Thus, GO-Y022 showed a minor impact on the suppressive ability of Tregs.

### 3.4 GO-Y022-treatment showed a substantial increase in the glycolysis of gastric cancer cells

**Figure 4:**
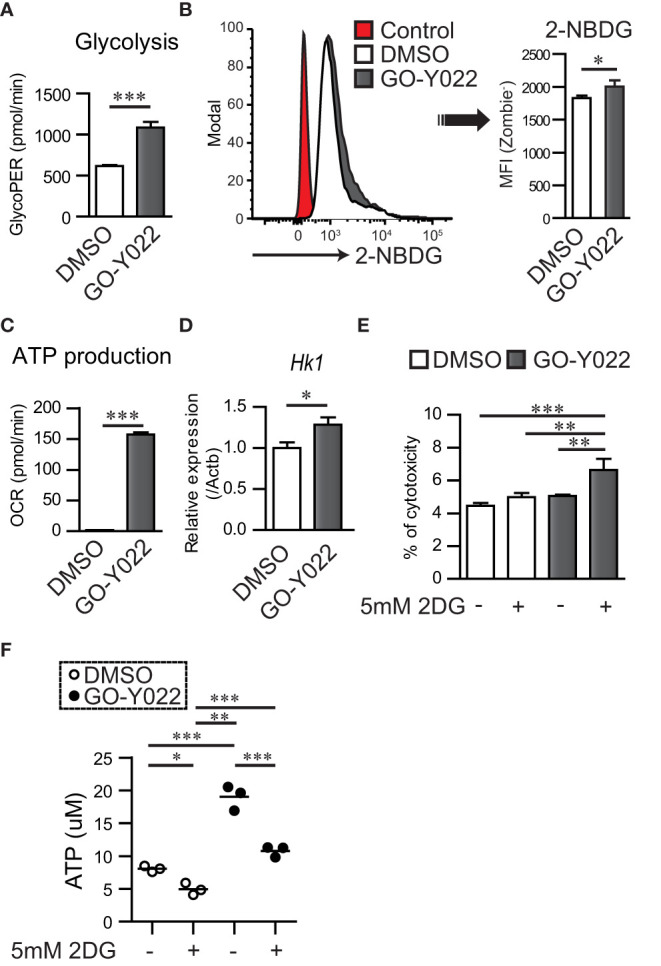
GO-Y022 treatment controls mitochondrial ATP production and cancer metabolism. **(A)** Glycolysis measured using the glycolytic rate test. Glycolysis was calculated after Rotenon/Antimycin A treatment based on the rate of OCR in gastric tumor SH-10-TC cells. White bar indicates or 0.007 μM DMSO treatment; black bar indicates 5 μM GO-Y022 treatment for 3 (h) **(B)** Glucose uptake of gastric tumor SH-10-TC cells measured using the 2-NBDG. Mean Fluorescence Intensity (MFI) was calculated by flow cytometric analysis. Red: Control (0.007 μM DMSO-treatment for 3h), White: 0.007 μM DMSO-treatment for 3h and then added 2-NBDG, Black: 5 μM GO-Y022-treatment for 3h and then added 2-NBDG. **(C)** ATP production was calculated based on the rate of OCR using the mitochondrial stress test. ATP production was calculated after olygomycin treatment based on the rate of OCR in gastric tumor SH-10-TC. White bar: 0.007 μM DMSO treatment; black bar: 5 μM GO-Y022 treatment for 3 (h) **(D)** Relative gene expression of 0.007 μM DMSO or 5 μM GO-Y022 treated SH-10-TC gastric tumor cells for 3 (h) Each genes expression normalized by using Hprt1, then DMSO treatment was set as “1.” **(E)** A cytotoxicity assay was performed using cultured SH-10-TC (h) White bar represents 0.007 μM DMSO treatment; black bar represents 5 μM GO-Y022 treatment. Data represent at least three independent experiments (mean + standard deviation, triplicate samples). **(F)** Extra cellular ATP concentration in SH-10-TC treated for 24h. White circle represents 0.007μM DMSO treatment; Black circle represents 5 μM GO-Y022 treatment. Data represent three independent experiments (mean, triplicate samples). Student’s t-test **(A–D)** or one-way ANOVA with *post-hoc* Tukey’s multiple comparison test **(E, F)** was applied. Statistical significance was set at *p* < 0.05; *p < 0.05, **p < 0.01, and ***p < 0.001.

GO-Y022, which can be derived from curry, can shrink gastric cancer cells when consumed in mouse model (12). To understand the mechanism of regulation of gastric tumor carcinogenesis, proliferation, and cell survival by GO-Y022, we focused on glucose metabolism, which is crucial in carcinogenesis, proliferation, and cell survival of gastric cancer (13). We previously demonstrated that 5 μM GO-Y022 treatment prevents cell proliferation and induces apoptosis of the gastric tumor cell line SH-10-TC (12). However, for the first 3 h, 5 μM GO-Y022-treatment did not induce cell death in gastric tumor cells (**Figure S7**). Hence, we hypothesized that 5 μM GO-Y022 treatment could induce rapid metabolic changes, prevent gastric tumor cell proliferation and induce apoptosis. To investigate this hypothesis, we first performed a glycolytic rate assay in gastric tumor cell lines using a Seahorse Bioscience XF^®^96 Extracellular Flux Analyzer. Glycolysis for DMSO treated SH-10-TC, was 618.2 ± 12.56 pmol/min, whereas for GO-Y022-treatment was 1085.2 ± 68.5 pmol/min (**Figure 4A**), which depended on the cellular adenosine triphosphate (ATP) demand. GO-Y022-treated gastric tumor GCIY cells also demonstrated a notable increase in glycolysis compared to those treated with DMSO alone (DMSO; 667.6 ± 8.12 pmol/min, GO-Y022; 962.8 ± 63.5 pmol/min) (**Figure S8A**). We also found that glucose uptake of SH-10-TC was higher after 5 μM GO-Y022-treatment as compared to DMSO-treatment (DMSO; 1827.3 ± 41.58, GO-Y022; 2003.3 ± 98.79) (**Figure 4B**). Moreover, as mitochondrial ATP production induces cell death by releasing pro-apoptotic factors (24), we next performed a mitochondrial stress test in gastric tumor cell lines. For DMSO treated SH-10-TC cells, it was 1.77 ± 0.23 pmol/min, whereas for GO-Y022-treatment was 157.3 ± 3.38 pmol/min (**Figure 4C**). We also found that GO-Y022 treated gastric tumor GCIY cells demonstrated a notable increase in mitochondrial ATP production compared to those treated with DMSO alone (DMSO; 0.97 ± 0.26 pmol/min, GO-Y022; 42.9 ± 1.90 pmol/min) (**Figure S8B**). Moreover, GO-Y022 treated GCIY and SH-10-TC cells showed 1.26- and 1.28-fold higher expression of hexokinase 1 (*Hk1*), respectively, which plays crucial roles in glycolysis and ATP synthesis (**Figures 4D**, **S8C**). Thus, GO-Y022-treatment enhanced more glycolysis in gastric tumor cell lines. Next, we used 2DG, a glucose analog, to inhibit glycolysis and found that 2DG-treatment (5 mM) has a minor effect in inducing cytotoxicity in SH-10-TC cells (Medium alone: 4.46 ± 0.16%, 2DG: 4.99 ± 0.25%) (**Figure 4E**). Although 5 uM GO-Y022-treatment has a little effect inducing cytotoxicity in SH-10-TC cells (5.05 ± 0.07%), combination with 2DG-tremtment (5 mM) showed strongest effects to induced cytotoxicity (6.62 ± 0.67%) (**Figure 4E**). GCIY also showed a similar effect (Medium alone: 15.675 ± 0.21%, 2DG: 17.58 ± 0.3%, GO-Y022: 17.8 ± 0.16%, GO-Y022 + 2DG: 20.0 ± 0.03%) (**Figure S8D**). We also found that the extracellular ATP concentration was significantly increased when GO-Y022-treated in SH-10-TC, and that increase was countered in the presence of 2DG (**Figure 4F**). When we examined whether a low dose of DMSO affected the metabolism of tumor cells (25), 0.007 μM DMSO treatment did not impact the ATP production, L-lactate production (**Figures S9A–C**) and metabolism-related gene expression (**Figure S9D**) of gastric tumor cell. These results suggest that GO-Y022-treated gastric cancer cells have a higher demand for glycolysis to survive.

### 3.5 The combination of pyrolyzed deketene curcumin and 2-DG has shown potential for a synergistic effect in gastric tumor therapy by inducing metabolic changes

**Figure 5:**
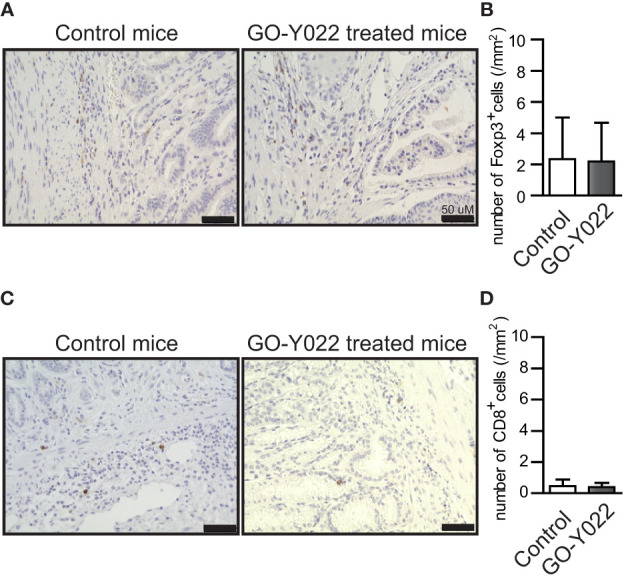
GO-Y022 does not significantly affect the Foxp3^+^ Treg population in the gastric tumor microenvironment. **(A, C)** Immunohistochemistry of Foxp3 **(A)** or CD8 **(C)** levels in gastric tumor sites. Representative images from three mice (n = 3). The original magnification is ×20. **(B, D)** Absolute number of Foxp3^+^ Tregs or CD8^+^ cells in tumor areas (mm^2^). Student’s t-test was used **(B, D)**. The graph shows the mean and standard deviation.

GO-Y022-treated Gan mice demonstrated reduced gastric cancer carcinogenesis compared to DMSO-treated Gan mice (12). Using the paraffin slides of gastric cancer, no significant changes were noted in the ratio of Foxp3^+^ Tregs in the tumor when Gan mice were fed a high-fat diet with GO-Y022 (number of Tregs in the tumor: control mice = 2.34 ± 2.65/mm^2^, GO-Y022-treated mice = 2.20 ± 2.47/mm^2^, p-value = 0.9487) (**Figures 5A, B**). Regarding the effector cells (CD8^+^ cells) in the slides, the number of effector cells in the tumor was not significantly different compared to the control group (number of CD8 T cells in the tumor areas (mm^2^): control mice = 0.46 ± 0.42/mm^2^, GO-Y022-treated mice = 0.40 ± 0.27/mm^2^, p-value = 0.8472) (**Figures 5C, D**).

To clarify the reason for the minor impact of GO-Y022-treatment on anti-tumor immunity in the gastric tumor microenvironment, we focused on the metabolic changes in the gastric tumor cells (**Figure 4C**). GO-Y022-treated gastric tumor cells demonstrated increased glycolysis and ATP production compared with DMSO-treated gastric tumor cells, which contributed to the production of many liquid factors, including TGF-β and L-lactate. First, we found that TGF-β production from gastric tumor SH-10-TC cells was significantly reduced in the presence of GO-Y022 treatment (**Figure S10A**). Additionally, 2DG-treatment prevented TGF-β production, which can be reduced further in the presence of GO-Y022 (**Figure S10A**). However, GO-Y022-treatment increased L-lactate production from gastric tumor cells (4.675 ± 0.045 mM) compared to the untreated control group (4.159 ± 0.064 mM) (**Figure S10B**). We also found that 2DG-treatment prevented L-lactate production even in the presence of GO-Y022 (**Figure S10B**). Though we demonstrated that GO-Y022 inhibits Treg generation in response to TGF-β (**Figures 1D–F**), large amounts of L-lactate diminished the inhibitory role of GO-Y022 in Treg generation *in vitro* (**Figures S10C, D**). On the contrary, administering of 2DG inhibited TGF-β-induced Tregs generation even in the presence of L-lactate (**Figure S11**). Therefore, we speculated that the L-lactate production from GO-Y022-treated gastric cancer cells overcomes the inhibitory roles of Treg generation caused by GO-Y022, and the combination with 2DG promotes starvation-induced tumor cell death and inhibit Tregs’ generation in the tumor microenvironment.

**Figure 6:**
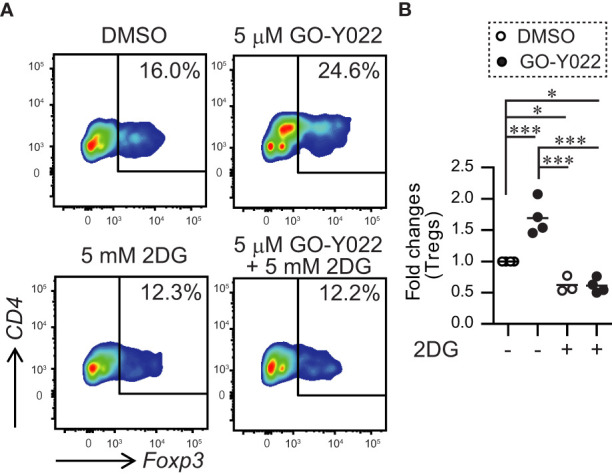
GO-Y022 treatment of human CD4+ T cells together with gastric tumor cells. **(A, B)** The frequency of Foxp3^+^ Tregs in the entire CD4^+^ cell population. Naïve CD4^+^T cells from human PBMCs (1 × 10^5^ cells) and SH-10-TC (3 × 10^4^ cells) were co-cultured in the presence of CD3/28 Dynabeads with or without 5mM 2DG for 48 **(h)** The data show representative density plots **(A)**. Statistical analyses of the percentage of Foxp3^+^Tregs were compared between 0.25 μM GO-Y022-treated and 0.007 μM DMSO-treated cultured CD4^+^ T cells with or without 2DG. Data were pooled from three independent experiments **(B)**. One-way ANOVA with *post-hoc* Tukey’s multiple comparison test was applied. Statistical significance was set at *p* < 0.05; *p < 0.05, and ***p < 0.001.

Next, we performed a co-culture experiment using gastric tumor cells and human naïve CD4^+^ T cells to examine the ability of Foxp3^+^ Tregs generation in the tumor microenvironment. As expected, human naïve CD4^+^ T cells induced Foxp3^+^ Tregs generation in the tumor microenvironment (**Figures 6A, B**). Since GO-Y022-treated gastric tumor cells produce large amounts of L-lactate, GO-Y022 in this co-culture system did not prevent Foxp3^+^ Treg generation of in the tumor microenvironment (**Figures 6A, B**). L-lactate (tumor metabolite) has been known to promote TGF-β-induced Treg generation in a dose dependent manner (26). These results suggest that GO-Y022-treatment in the gastric tumor microenvironment does not promote anti-tumor immunity. Of note, 2DG treatment significantly prevented Foxp3^+^ Treg generation of even in the presence of GO-Y022 (**Figures 6A, B**). We suggest that GO-Y022 cooperates with 2DG-treatment strongly to prevent TGF-β and L-lactate production, therefore Foxp3^+^Treg generation of in the gastric tumor microenvironment was significantly reduced in the presence of GO-Y022 and 2DG. Thus, combining GO-Y022 treatment with inhibition of glucose metabolism could be used as a potential gastric tumor therapy.

## 4 Discussion

Curcumin provides various health benefits, including antitumor immunity. Mice with mammary carcinomas fed with curcumin showed decreased tumor volume and Treg population in draining lymph nodes and tumors (27). A previous study showed that 10-μM curcumin inhibited TGF-β-induced Foxp3+ Tregs *in vitro* (28). A clinical trial showed that curcumin intake (3000 mg curcumin per day) reduced Foxp3+ Tregs in peripheral mononuclear cells (29). In our previous study, we found that treatment with low-dose curcumin (5 mg/kg) did not reduce the tumor size and Foxp3+ Tregs population in the tumor microenvironment in B16-F10 melanoma-bearing mice (30). Therefore, high-dose curcumin is expected to develop antitumor immunity.

To overcome this problem, we focused on the deketene curcumin analog, GO-Y022. GO-Y022 could be generated by the pyrolysis of curcumin, which occurs during the heating process in cooking curry (31). GO-Y022 has a strong antitumor effect and induces G2 arrest in melanoma cells (31). In gastric tumor cell lines, the average IC50 of GO-Y022 is 5-fold lesser than that of curcumin (12). K19-Wnt1/C2mE (Gan) mice with gastric cancers treated with high-dose GO-Y022 (1,250 mg/kg) by natural feeding showed tumor shrinkage, and no problems were observed in their health. Although the effective and safe doses of GO-Y022 treatment are still unclear, curry pastes, which contain GO-Y022, could be an antitumor food component. Therefore, more efficient antigastric tumor effects and antitumor immunity could be obtained with low-dose GO-Y022.

In this study, GO-Y022 inhibited Foxp3+ Treg generation *in vitro*. For the molecular mechanisms of Treg generation, the T cell receptor–calcium–calcineurin pathway activates NFATc1 enrichment on the Foxp3 promoter and conserved CNS1 regions and positively regulates Foxp3 gene expression (32). In NFATc1 deficiency, the Foxp3+ Treg generation fails in response to TGF-β (33). Thus, inhibiting NFATc1 enrichment of Foxp3 gene regulatory elements using GO-Y022 is a key mechanism for preventing Treg generation. Although the Foxp3 reporter plasmid did not contain the CNS1 regions, this study revealed that GO-Y022 prevents Foxp3 promoter activity in response to Ionomycin (NFAT activator) (**Figure 2D**). Furthermore, TGF-β signal molecule Smad plays a crucial role in induced Foxp3+ Treg cooperated with NFATc1 (20). However, GO-Y022 treatment did not affect Smad signaling in cultured naïve CD4+ T cells in response to TGF-β (**Figures 2A, B**). Therefore, GO-Y022 treatment exclusively controls NFATc1 enrichment on the Foxp3 promoter and CNS1 regions.

Regarding the stability of Tregs, GO-Y022-treated Tregs did not reduce the expression of Foxp3 (**Figures 3B, C**). Additionally, the mean fluorescence intensity (MFI) of Foxp3 in Tregs reflected their suppressive ability (30). However, no difference was observed between DMSO- and GO-Y022-treated Tregs (**Figures 3B–F**). CD4+ CD25+ Tregs inhibit CD8+ T cell-mediated tumor rejection (23). Although CD4+ CD25+ Tregs suppressed the proliferation of CD8+ T cells, GO-Y022 treatment had little impact on the suppressive ability of Tregs against the proliferation of CD8+ T cells. In Gan mice, the numbers of CD8+ T cells in the gastric tumor sites were not different, even if the mice were fed a high-fat diet with high-dose GO-Y022 (**Figures 5C, D**). Therefore, whether GO-Y022 treatment affects the CD8+ T cell tumor inhibition is still unclear due to IFN-γ production from the CD8+ T cells. However, we found GO-Y022 has greater potential to control Treg generation in response to TGF-β.

Glucose metabolism in gastric cancers plays a crucial role in carcinogenesis, invasion, and metastasis (34). GO-Y022 treatment enhanced Hk1 expression in gastric tumor cells (**Figure 4D**), thus promoting glycolysis, proliferation, and lymphatic metastasis (35). The high Hk1 expression in gastric tumors showed poor prognosis (36). We further demonstrated that GO-Y022 treatment with glycolysis inhibition (treated with 2DG) induced gastric tumor cell death (**Figures 4E**, **S8D**).

L-lactate in the gastric tumor microenvironment prevents antitumor immunity (37). Mechanistically, L-lactate promotes TGF-β-induced Tregs (14) and their proliferation and suppressive ability in the tumor microenvironment (38). In human naïve CD4+ T cells, 10 mM L-lactate (but not purine and pyrimidine) promotes Treg generation in response to TGF-β (26). TGF-β-induced Treg generation was inhibited by GO-Y022 treatment, but Treg generation was not inhibited in the presence of 100-μM L-lactate (**Figures S10C, D**). Furthermore, the level of ATP release from SH-10-TC cells was significantly increased by GO-Y022 treatment (**Figure 4F**). Although ATP inhibits Treg generation and function (39), we speculate that these functions are countered by TGF-β and L-lactate production from gastric tumor cells (**Figures S10B–D**). L-lactate is a metabolic product of glycolysis. Our findings showed that glycolysis was recovered by GO-Y022 treatment in the gastric tumor cells compared with the control (**Figure 4A**). In case of curcumin treatment, L-lactate production was reduced in a dose-independent manner (2.5–20 μM) in different tumor cells (40). Gan mice treated with a high-fat diet with GO-Y022 showed shrinkage of the tumor size (12), but the number of Foxp3+ Tregs per tumor area (mm^3^) was not different from that of the control mice (**Figure 5B**). Glycolysis promotes gastric tumor cell proliferation (41). We speculated that the activation of glycolysis in gastric tumor cells with GO-Y022 and the resulting L-lactate production could conversely cancel the inhibition of Treg generation with GO-Y022.

**Figure 7:**
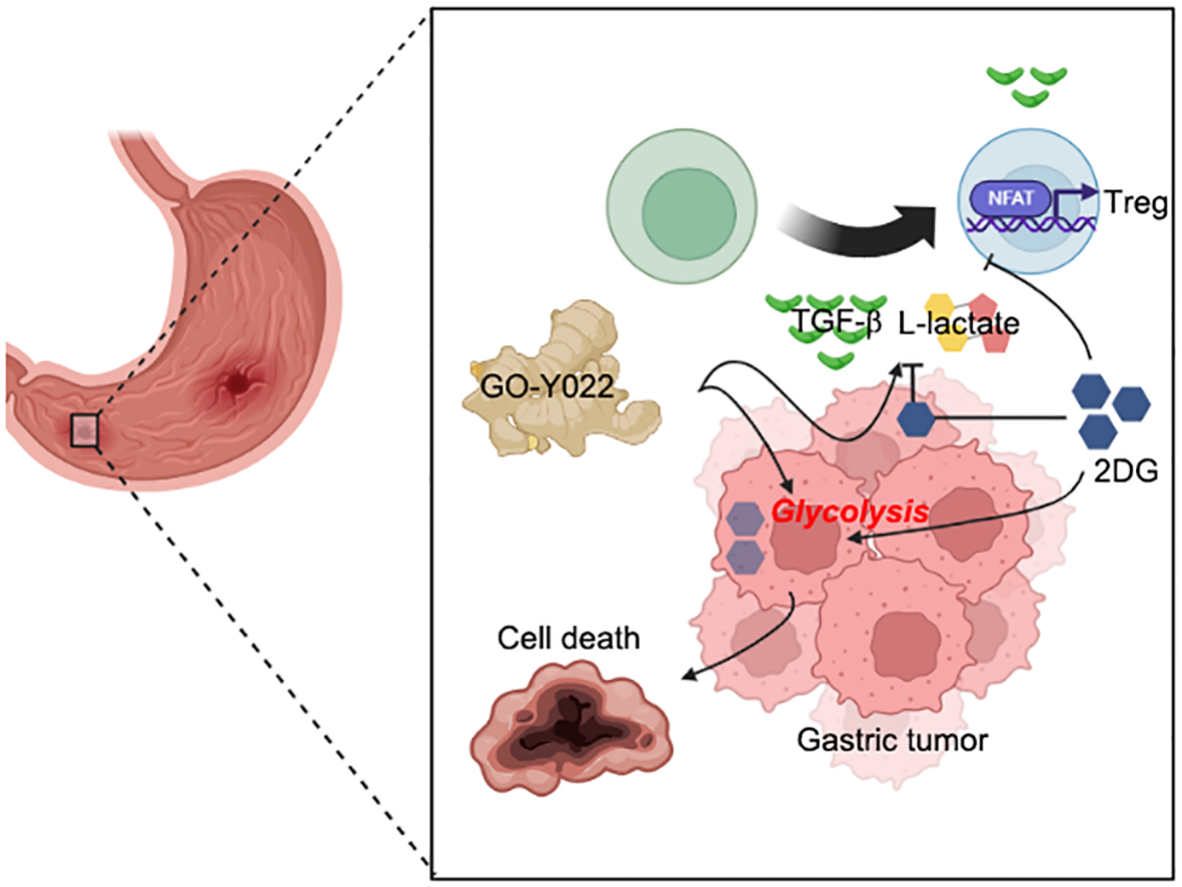
Coadministration of GO-Y022 and 2DG. The effect of GO-Y022: GO-Y022 treatment enhances glycolysis in gastric tumor cells and helps produce a large amount of TGF-β and L-lactate. TGF-β and L-lactate strongly induce suppressive function, Treg generation, and its function in the tumor. The effect of 2DG: 2DG inhibits glycolysis and prevents TGF-β and L-lactate production. 2DG treatment also inhibits Treg generation in response to TGF-β and L-lactate. The synergistic effect of GO-Y022 plus 2DG: the amount of 2DG uptake could be much higher in the gastric tumor cells in the presence of GO-Y022, resulting in further glycolysis inhibition. Therefore, coadministration of GO-Y022 and 2DG strongly induces tumor cell death and inhibits TGF-β and L-lactate production. Coadministration of GO-Y022 and 2DG showed immunological inhibition of Treg generation in the tumor.

2DG inhibits gastric tumor cell growth and prevents lactate production by preventing glycolysis (42). 2DG treatment overcomes TNF-related apoptosis-inducing ligand resistance in gastric tumor cells (MGC803 and SGC7901) (43). 2DG also prevents L-lactate production from cancer cells (44). A clinical trial of 2DG showed that patients with tumors receiving 45 mg/kg 2DG orally for up to 2 weeks showed no toxicity and glucose uptake reduction in tumor cells (45). 2DG inhibits TGF-β-induced Tregs (46) and enhances antitumor immunity (47). Our coculture system demonstrated that GO-Y022 treatment in the tumor microenvironment enhanced Treg generation, but coadministering of 2DG with GO-Y022 counteracted this effect (**Figures 6A, B**). However, high-dose 2DG (<8,000 mg/kg, p.o.) induced acute toxicity and cardiorespiratory effects (48). These authors also demonstrated that the LD50 of 2DG in mice by intravenous injection was 8,000 mg/kg. The therapeutic dose of 2DG in humans has been reported to be 250 or 90 mg/kg per day (49). Additionally, in most mice, CD4+ T cells died in the presence of 10 mM 2DG despite the presence of 2 ng/ml TGF-β (data not shown). As 2DG treatment (5 mM = 820.8 mg/L) inhibits glycolysis in the gastric tumor cells, L-lactate production was reduced in the presence of GO-Y022 (**Figure S10B**). L-lactate promotes Treg generation and its function in response to TGF-β (14, 26). Foxp3+ Treg generation was inhibited with 5 mM 2DG treatment in the presence of GO-Y022 (**Figures 6A, B**). Although neither GO-Y022 treatment (**Figures 3B, C, F, G**) nor 2DG (50) demonstrated a minor effect on the stability of Foxp3+ Tregs *in vitro*, combined treatment with GO-Y022 and 2DG could affect the Foxp3+ Treg generation and stability in the tumor microenvironment. GO-Y022 treatment would help elevate glucose uptake in tumor cells in patients with gastric tumors. Therefore, combined treatment with GO-Y022 and 2DG could have synergic antitumor effects (**Figure 7**). Additionally, GO-Y022 treatment could reduce the amount of 2DG administration with curcumin (51). Future studies are needed to investigate whether GO-Y022 enhances CD8+ T cell-mediated tumor rejection.

## Data availability statement

The raw data supporting the conclusions of this article will be made available by the authors, without undue reservation.

## Ethics statement

Human PBMCs (Informed consent obtained) were purchased from BioIVT (NY). This study was reviewed and approved by Tohoku University. The animal study was reviewed and approved by Akita University and NIDCR.

## Author contributions

TM conceived of and directed this study, designed and performed most of the experiments, analyzed the data, and wrote this manuscript. HM and Y-JL performed the experiments and analyzed the data. JG, MI, TY, WC and YO provided critical suggestions and materials. HS supervised the experiments and contributed to the editing of this manuscript. All authors contributed to the article and approved the submitted version.
