# Supplementary material for: Pyrolyzed deketene curcumin controls regulatory T cell generation and gastric cancer metabolism cooperate with 2-deoxy-d-glucose
Source: Front Immunol. 2023 Feb 6;14:1049713. doi: 10.3389/fimmu.2023.1049713 (PMC9939626; doi:10.3389/fimmu.2023.1049713)
Supplement: Supplementary file 1 [file DataSheet_1.docx]

| **(For Taqman)** | **Cat#** |
| --- | --- |
| Hprt1 | Mm00446968_m1 |
| Foxp3 | Mm00475162_m1 |
| ACTB | Hs99999903_m1 |
| HK1 | Hs00175976_m1 |
| INHBA | Hs01081598_m1 |
| MYC | Hs00153408_m1 |
| GLUT1 | Hs00892681_m1 |

**Supplementary Table 1. List of primers used in this study**


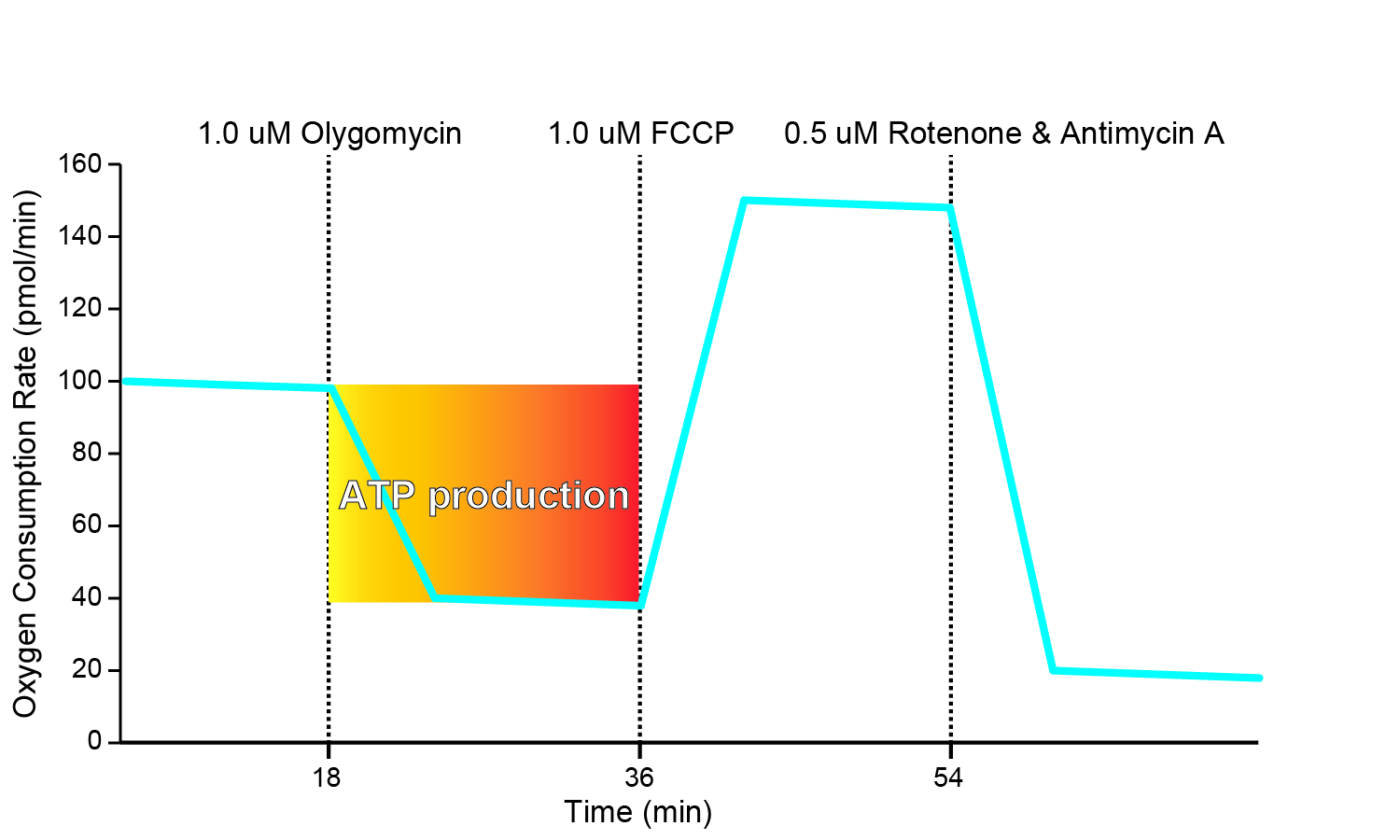


**Supplementary Figure 1. Diagram of Seahorse XF cell Mito Stress Test Profile**

ATP production (Orange-filled) was calculated based on Oxygen consumption Rate (OCR) after Olygomycin treatment.


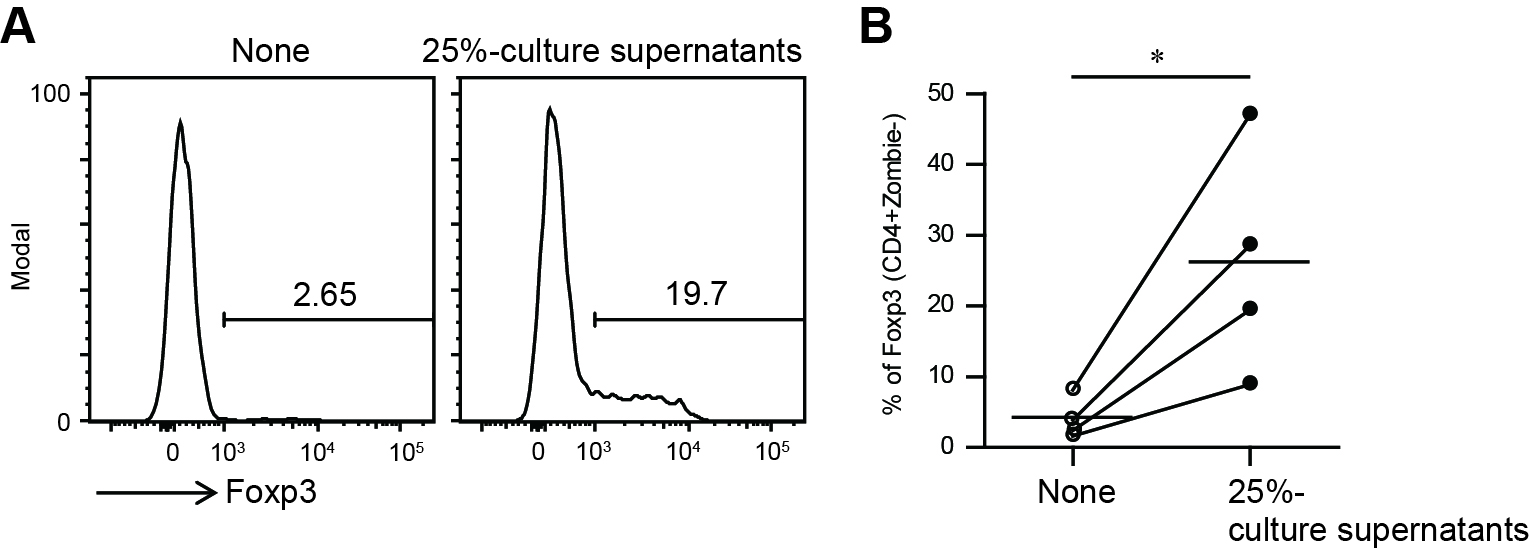


**Supplementary Figure 2. Supernatants from gastric tumor cell lines were used to induce Foxp3^+^ Tregs**

Splenic naïve CD4^+^ T cells were cultured in the presence or absence of the culture supernatant of the gastric tumor cell line SH-10-TC. After 72 h, Fluorescence activated cell sorting (FACS) analysis was performed. (**A**) Representative FACS histogram. Percentages of Foxp3^+^ Tregs in the CD4^+^Zombie^-^ population. (**B**) Data were pooled from four independent experiments. The circles indicate independent experiments and the horizontal bars represent the mean. Students’ t-test was employed. Statistical significance was set at *p* < 0.05; *p < 0.05.


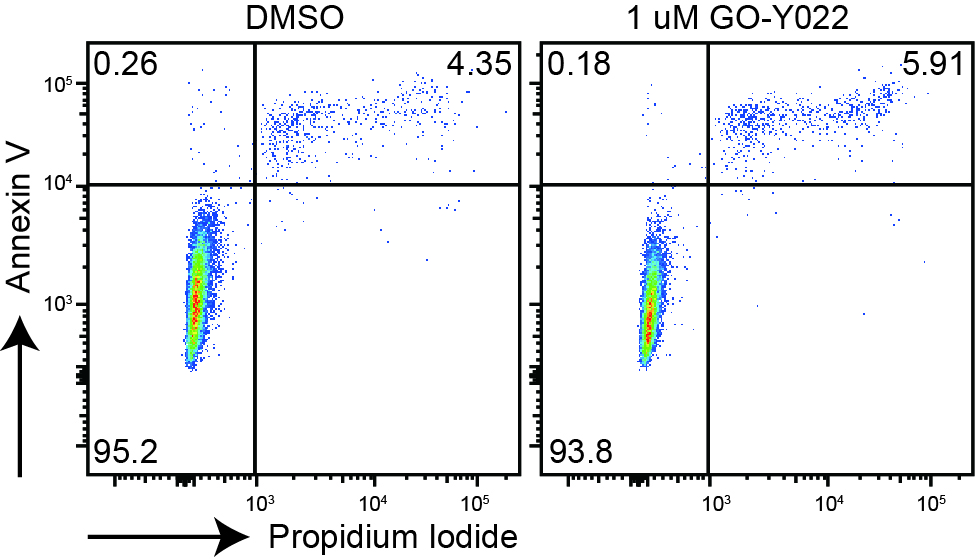


**Supplementary Figure 3. GO-Y022- did not induce apoptotic cell death in EL4/LAF lymphoma cells after 24 h**

Relative Annexin V and propidium iodide staining in cultured EL4/LAF lymphoma cells for 24 h. Data show one representative experiment of at least two independent experiments. 0.007 μM DMSO was used for control.


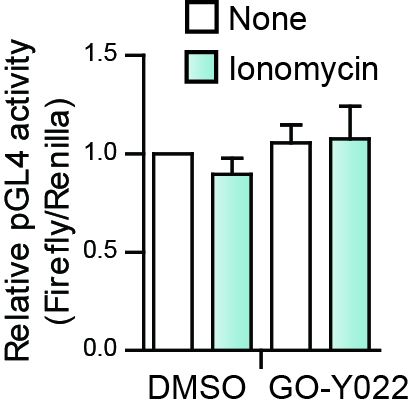


**Supplementary Figure 4. Control of Foxp3 reporter promoter assay using pGL4**

The pGL4-basic vector electroporated in EL4 lymphoma cells was used as a control for the Foxp3-reporter assay. The EL4 cells were cultured with 1 μM GO-Y022 or 0.007μM DMSO in the presence or absence of PMA + ionomycin for 6 h. DMSO without the PMA + ionomycin condition was set as “1.” Data shown represent one of at three independent experiments (triplicate data, mean + standard deviation).


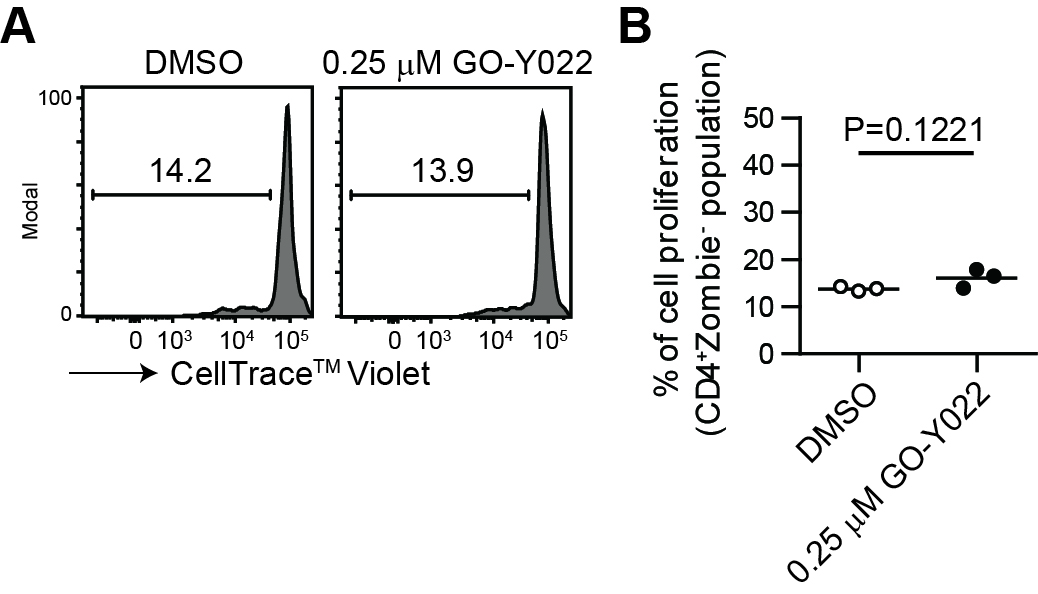


**Supplementary Figure 5. GO-Y022-treatment dose not inhibit CD4^+^CD25^+^Tregs’ proliferation**

(**A**, **B**) The frequency of CellTrave Violet-positive population in CD4^+^ Zombie^-^ cell population. Purified splenic CD4^+^CD25^+^ Tregs were labeled CellTrace^TM^ Violet (Thermo-Fisher Scientific, 1:1000 dilution in PBS, 37°C for 20 min in dark according to the manufacture’s institution) and then cultured with 1 μg/mL plate-bound anti-CD3, 1 μg/ml soluble anti-CD28 and 10 ng/mL human IL-2 with 0.25 μM GO-Y022 or 0.007 μM DMSO for 3 days. Data represent at three independent experiments. The graph shows the mean. Student’s t-test (**B**) was employed.


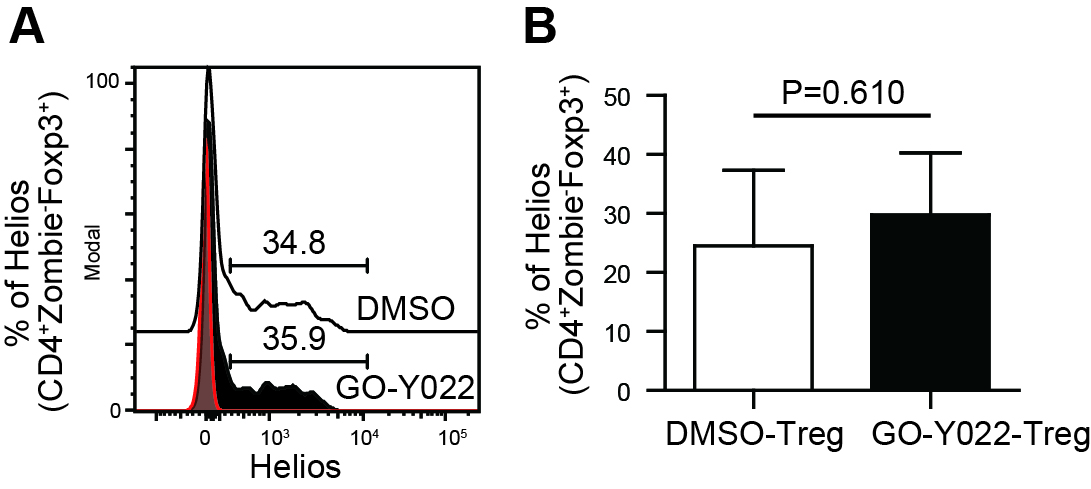


**Supplementary Figure 6. Helios expression in cultured CD4^+^ CD25^+^ Tregs**

(**A**, **B**) The frequency of Helios-positive population in CD4^+^ Zombie^-^ Foxp3^+^ cell population. Purified splenic CD4^+^ CD25^+^ Tregs were cultured with 1 μg/mL plate-bound anti-CD3, 1 μg/ml soluble anti-CD28 and 10 ng/mL human IL-2 with 0.25 μM GO-Y022 (Black filled) or 0.007 μM DMSO (White filled) for 3 days. Red filled is isotype control. Data represent at three independent experiments. The graph shows the mean. Student’s t-test (**B**) was employed.


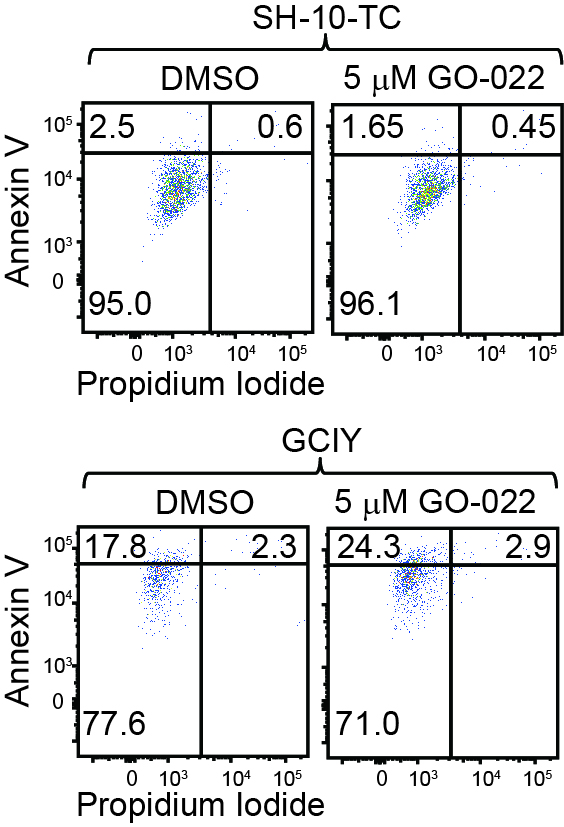


**Supplementary Figure 7. GO-Y022- did not induce apoptotic cell death in gastric cancer cells after 3 h**

Relative Annexin V and propidium iodide staining in cultured SH-10-TC and GCIY gastric tumor cells for 3 h. Data show one representative experiment of at least two independent experiments. 0.007 μM DMSO was used for control.


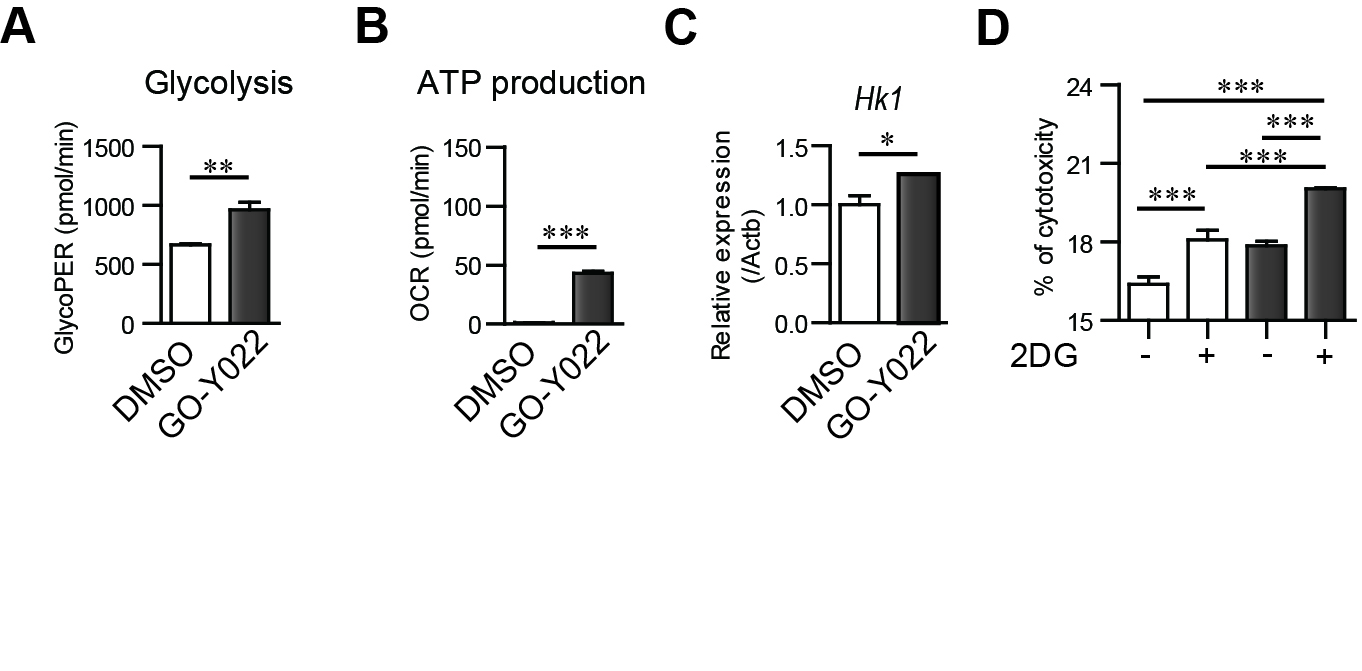


**Supplementary Figure 8. Metabolic phenotype of GCIY in the presence of GO-Y022**

(**A**) Glycolysis was measured using the glycolytic rate test. White bar indicates DMSO treatment; black bar indicates 5 μM GO-Y022 treatment for 3 h. (**B**) ATP production was calculated based on the rate of OCR using the mitochondrial stress test. White bar indicates 0.007 μM DMSO treatment; black bar indicates 5 μM GO-Y022 treatment for 3 h. (**C**) Relative gene expression of DMSO- or 5 μM GO-Y022 treated GCIY gastric tumor cells for 3 h. 0.007 μM DMSO treatment was set as “1.” (**D**) A cytotoxicity assay was performed using cultured GCIY for 24 h. White bar indicates 0.014μM DMSO treatment; black bar indicates 10 μM GO-Y022 treatment. Data represent at least three independent experiments (mean + standard deviation, triplicate samples). Student’s t-test was applied. Statistical significance was set at *p* < 0.05; *p < 0.05, **p < 0.01, and ***p < 0.001.

**
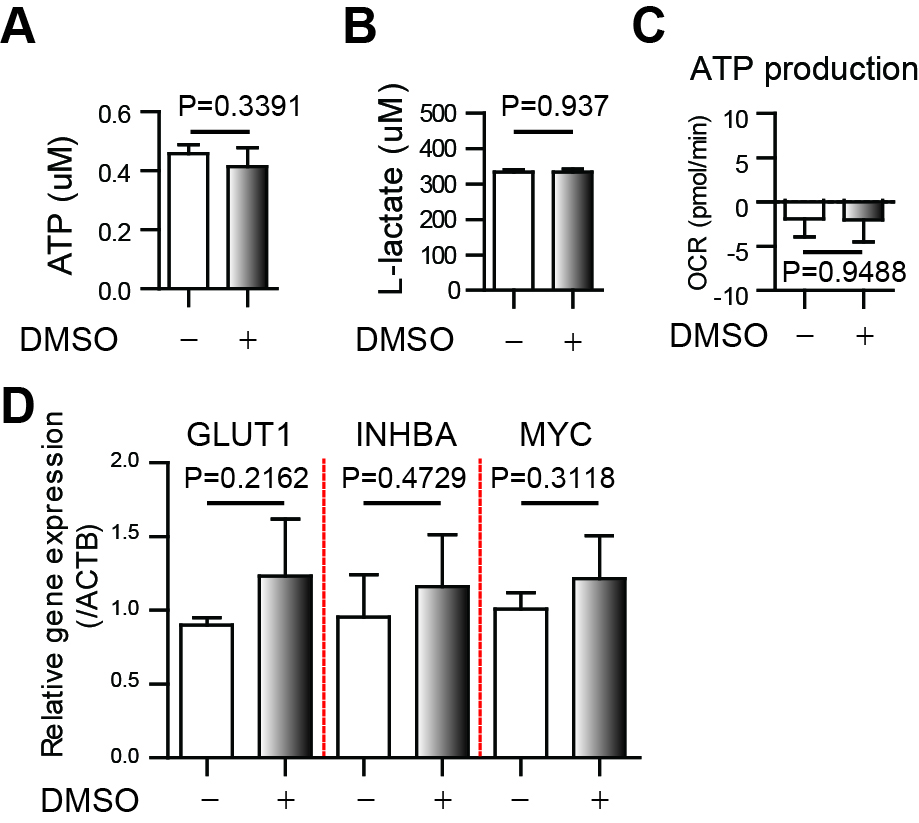
**

**Supplementary Figure 9. Low dose of DMSO showed no impact of metabolisms in gastric tumor cells.**

(**A**, **B**) Extra cellular ATP (**A**) and L-lactate (**B**) concentration cultured SH-10-TC for 24h. White bar represents medium alone; Black circle represents 0.007μM DMSO treatment. Data represent three independent experiments (mean, triplicate samples). (**C**) ATP production was calculated based on the rate of OCR using the mitochondrial stress test. ATP production was calculated after Olygomycin treatment based on the rate of OCR in gastric tumor SH-10-TC. White bar: medium alone; black bar: 0.007 μM DMSO treatment for 3 h. Data represent two independent experiments (mean, triplicate samples). (**D**) Relative genes’ expression of medium alone or 0.007 μM DMSO-treated SH-10-TC gastric tumor cells for 24 h. Data represent two independent experiments (mean, triplicate samples). Student’s t-test (**A**–**D**) was applied. Statistical significance was set at p < 0.05; ∗p < 0.05, ∗∗p < 0.01, and ∗∗∗p < 0.001.


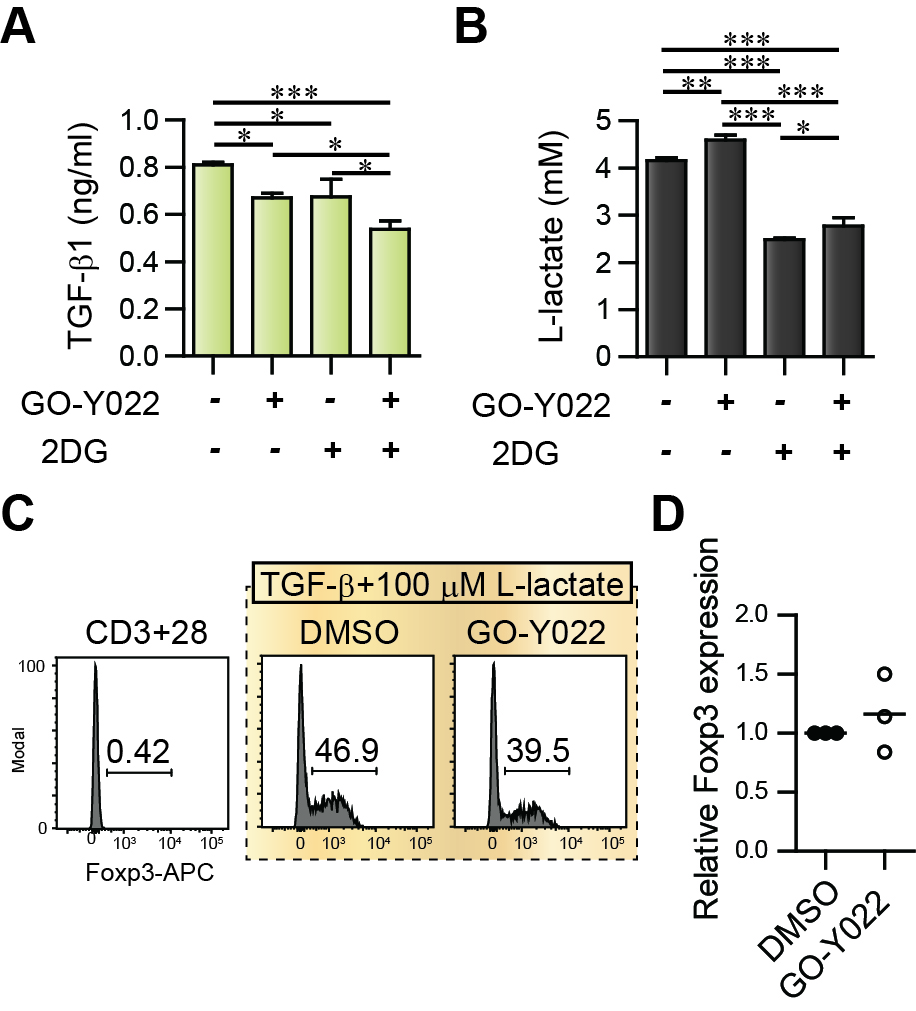


**Supplementary Figure 10. L-lactate + TGF-**β**1 treatment diminished GO-Y022-inhibited Treg generation *in vitro***

(**A, B**) Concentration of TGF-β1 (**A**) or L-lactate (**B**) in supernatants from gastric tumor SH-10-TC cells; 5 μM GO-Y022 or 5 mM 2DG treatment for 72 h. Data represent at least three independent experiments (mean + standard deviation, triplicate samples). One-way ANOVA with post-hoc Tukey’s multiple comparison test was applied. (**C**, **D**) Naïve CD4^+^ T cells were cultured in the presence or absence of TGF-β1+L-lactate with or without 0.25 μM GO-Y022 for 72 h. 0.007 μM DMSO was used for control. Data represent at least three independent experiments (**C**). Relative Foxp3^+^Treg expression in cultured wells. Percentage of Foxp3^+^Tregs in TGF-β1+L-lactate as set as “1” (**D**). Statistical significance was set at *p* < 0.05; *p < 0.05, **p < 0.01, and ***p < 0.001.


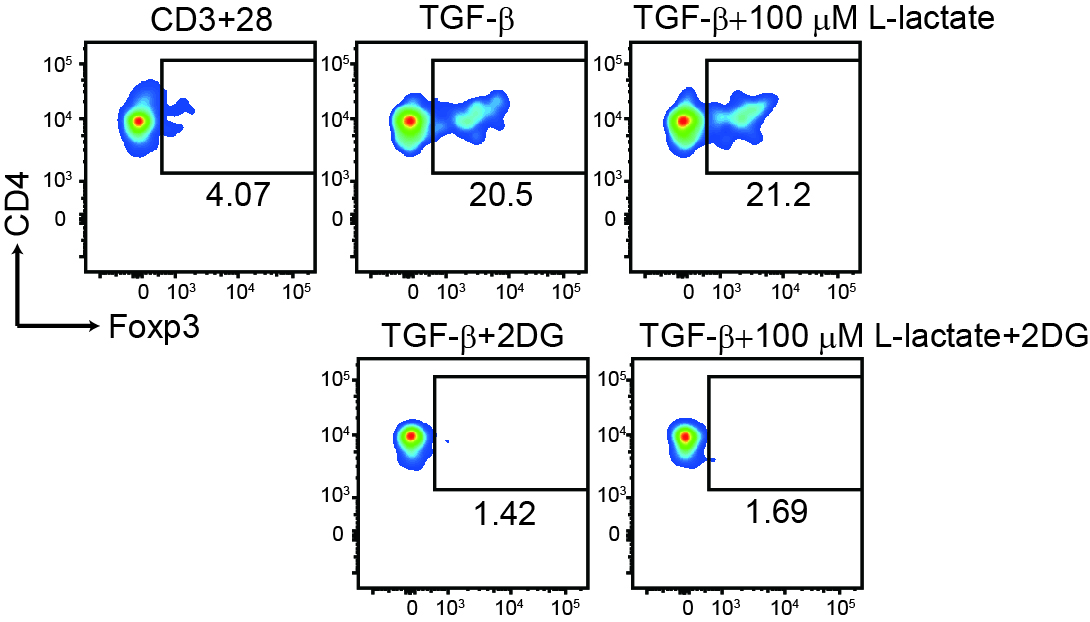


**Supplementary Figure 11. 2DG treatment prevents Treg generation *in vitro***

Splenic naïve CD4^+^ T cells were cultured in the presence or absence of 5mM 2DG. After 72 h, FACS analysis was performed. Representative FACS histogram. Percentages of Foxp3^+^ Tregs in the CD4^+^Zombie^-^ population. Data were representative at three independent experiments.
